# Comparative effectiveness research: Challenges for medical journals

**DOI:** 10.1186/1745-6215-11-45

**Published:** 2010-04-28

**Authors:** Harold C Sox, Mark Helfand, Jeremy Grimshaw, Kay Dickersin, The PLoS Medicine Editors, David Tovey, J André Knottnerus, Peter Tugwell

**Affiliations:** 1 Dartmouth Institute, Dartmouth Medical School, Hanover, New Hampshire, USA; 2 Portland VA Medical Center and Department of Medicine, Oregon Health & Science University, Portland, Oregon, USA; 3 Clinical Epidemiology Program, Ottawa Hospital Research Institute, Ottawa, Ontario, Canada; 4 Department of Epidemiology, Johns Hopkins Bloomberg School of Public Health, Baltimore, Maryland, USA; 5 The Cochrane Library, London, UK; 6 Department of General Practice, University of Maastricht, Maastricht, The Netherlands; 7 Departments of Medicine, and Epidemiology and Community Medicine, University of Ottawa, Ottawa, Ontario, Canada

## Abstract

Editors from a number of medical journals lay out principles for journals considering publication of Comparative Effectiveness Research (CER). In order to encourage dissemination of this editorial, this article is freely available in PLoS Medicine and will be also published in Medical Decision Making, Croatian Medical Journal, The Cochrane Library, Trials, The American Journal of Managed Care, and Journal of Clinical Epidemiology.

## 

In order to optimize health outcomes within the constraints of inevitably limited resources, low- and high-income countries alike require unbiased means of assessing health care interventions for their relative effectiveness. Such interventions include diagnostic tests and treatments (both established and newly developed) and implementation of health policy [1]. Likewise, health care professionals and patients need better information to inform health care decisions that require weighing benefits and risks in light of the patient's medical history and personal preferences.

Some countries and international organizations have recognized the need for such evidence and are already allocating funds for research to provide it [2]. The WHO Ministerial Summit in Mexico called for the establishment of support for a substantive and sustainable program of health systems research aligned with countries' priority needs and aimed at achieving internationally agreed-upon health-related development goals, including those contained in the United Nations Millennium Declaration [3]. The UK has established the National Institute for Health Research to commission and disseminate research that supports decision making by professionals, policy makers and patients and to ensure that the UK's health system, the National Health Service, has access to the best possible evidence to inform decisions and choices [4].

The US is now addressing similar goals with an initiative known as comparative effectiveness research (CER). In 2008, a report by the US Institute of Medicine (IOM) noted that patient care "should be based on the conscientious, explicit, and judicious use of current best evidence" [1]. In legislation that allocated US $1.1 billion in the US for CER on health care practices in 2009, the US Congress mandated that the IOM set national priorities for CER clinical topics. The IOM defined CER as "The generation and synthesis of evidence that compares the benefits and harms of alternative methods to prevent, diagnose, treat, and monitor a clinical condition, or to improve the delivery of care" [5]. The definition further stated that "The purpose of CER is to assist consumers, clinicians, purchasers, and policy makers to make informed decisions that will improve health care at both the individual and population levels."

To the authors and endorsers of the present Editorial, the potential value of research with these characteristics is self-evident. The challenge will be to realize the full potential of such research to improve health. Doing so will require assessing a heterogeneous body of evidence consisting of prospective randomized trials--including pragmatic trials--and observational research using data obtained in the course of regular practice. Hence, medical journals must use rigorous approaches, including but not limited to peer review by independent experts, to assess the limitations inherent in such research, such as missing data, incomplete follow-up, unmeasured biases, the potential role of chance, competing interests, and selective reporting of results.

Drawing on many years of collective experience in assessing these issues in the course of evaluating health research through peer review, we support the following principles and standards for CER.

## Principles of CER

• The principal goal of CER is to allow decision makers (patients, clinicians, purchasers, and policy makers) to make informed decisions on specific health practices. CER aims to identify and fill knowledge gaps that underlie uncertainties in practice.

• CER may provide information about individual and population benefits, harms, costs, and logistics of different policies or treatments.

• CER will apply to a broad range of interventions, tests, and strategies for prevention, care delivery, and quality of care

• CER should directly compare tests or active treatments--so-called head-to-head comparisons--of viable clinical alternatives within the current standard of practice (which in some cases may be no intervention).

• CER should primarily assess patient-relevant outcomes, but should also compare the economic implications of different approaches to prevention and care.

• CER should assist patients and physicians to choose between effective treatments. To this end, CER should identify patient characteristics that are associated with meaningful differences in outcomes. Researchers should collect and analyze the data necessary to achieve this goal according to a pre-specified plan that clearly indicates specific hypotheses and the methods to test them.

• The scope of CER includes new data, old data newly analyzed, and systematic reviews of existing research.

## Standards for the Conduct and Reporting of CER

• CER studies should follow the highest scientific standards for design, analysis, and interpretation and should adhere to reporting guidelines [6] that build upon initiatives to improve the quality and transparency of clinical science.

• Every CER study should have a research protocol, written in advance and addressing the key research question(s), methods, and planned analyses. Researchers should record all changes in the protocol. These protocols should be publicly accessible.

• Patients and other decision-makers should be involved in selecting and refining topics for CER.

• The study population for CER should be representative of clinical practice or the relevant public health practice.

• To increase transparency about selective publication, researchers should register CER studies before initiation, in a publicly available registry.

• To increase transparency about the practice of presenting post-hoc analyses as conclusive results, study registration should include a clear statement of study hypotheses, outcomes, and analysis plan.

• CER studies must undergo rigorous peer review by independent topical, methodological, and statistical experts.

• To ensure accessibility to the affected public and other researchers, journals (or other sites of publication) should make all CER studies freely available and archive them in a public repository, such as PubMed Central.

• Reports of CER must include a frank discussion of each study's limitations, including biases, confounding, and scope of applicability.

• Given the potential impact of CER on the profitability of the interventions being evaluated, researchers performing CER studies must commit to stringent and enforceable competing interest policies [7].

• Researchers, funders, and other contributors to a CER study must clearly state all relevant competing interests at the time of peer review, and publicly upon publication in any forum.

Medical journals are the primary evaluators and disseminators of peer-reviewed health research. As such, they must ready themselves to play a crucial role in advocating for CER, advancing CER methods and facilitating the translation of CER results into practice. Most importantly, journals and peer reviewers must do their part to ensure that CER, like all research with relevance to health, meets the highest scientific and ethical standards. They must therefore develop the methodological and statistical expertise to properly evaluate new or unfamiliar methods of health care research.

We recognize that CER has the potential to substantially improve decision-making about existing and new approaches to health care. To fulfill this potential, researchers must adopt stringent methods, and medical journals must hold them to high standards of ethics, scientific rigor, and reporting.

## Author information

HCS is Editor Emeritus, *Annals of Internal Medicine*. MH is Editor, *Medical Decision Making*. JG is Director of the Canadian Cochrane Center and Co-Editor in Chief, *Trials*. KD is Director, US Cochrane Center. DT is Editor in Chief, *The Cochrane Library*. AK and PT are Co-Editors, *Journal of Clinical Epidemiology*.

The *PLoS Medicine *Editors are Virginia Barbour, Jocalyn Clark, Susan Jones, Larry Peiperl, and Emma Veitch.

## Competing interests

The *PLoS Medicine *Editors' competing interests are at http://www.plosmedicine.org/static/editorsInterests.action. PLoS is funded partly through manuscript publication charges, but the *PLoS Medicine *Editors are paid a fixed salary (their salary is not linked to the number of papers published in the journal). The other authors have declared no competing interests.

## Authors' contributions

ICMJE criteria for authorship read and met: HCS MH JG KD VB JC SJ LP EV DT AK PT. Agree with the manuscript's results and conclusions: HCS MH JG KD VB JC SJ LP EV DT AK PT. Wrote the first draft of the paper: HCS. Contributed to the writing of the paper: HCS MH JG KD VB JC SJ LP EV DT AK PT.

## Funding

The *PLoS Medicine *Editors are each paid a salary by the Public Library of Science, and they contributed to this editorial during their salaried time. The other authors received no specific funding for this article.
